# Molecular Properties of β-Carotene Oxygenases and Their Potential in Industrial Production of Vitamin A and Its Derivatives

**DOI:** 10.3390/antiox11061180

**Published:** 2022-06-16

**Authors:** Kyung-Chul Shin, Min-Ju Seo, Yeong-Su Kim, Soo-Jin Yeom

**Affiliations:** 1 Department of Integrative Bioscience and Biotechnology, Konkuk University, Seoul 05029, Korea; hidex2@konkuk.ac.kr; 2 School of Biological Sciences and Technology, Chonnam National University, Gwangju 61186, Korea; 3 Wild Plants Industrialization Research Division, Baekdudaegan National Arboretum, Bonghwa 36209, Korea

**Keywords:** β-carotene oxygenase, vitamin A, retinal, enzyme engineering, metabolic engineering

## Abstract

β-Carotene 15,15′-oxygenase (BCO1) and β-carotene 9′,10′-oxygenase (BCO2) are potential producers of vitamin A derivatives, since they can catalyze the oxidative cleavage of dietary provitamin A carotenoids to retinoids and derivative such as apocarotenal. Retinoids are a class of chemical compounds that are vitamers of vitamin A or are chemically related to it, and are essential nutrients for humans and highly valuable in the food and cosmetics industries. β-carotene oxygenases (BCOs) from various organisms have been overexpressed in heterogeneous bacteria, such as *Escherichia coli*, and their biochemical properties have been studied. For the industrial production of retinal, there is a need for increased production of a retinal producer and biosynthesis of retinal using biocatalyst systems improved by enzyme engineering. The current review aims to discuss BCOs from animal, plants, and bacteria, and to elaborate on the recent progress in our understanding of their functions, biochemical properties, substrate specificity, and enzyme activities with respect to the production of retinoids in whole-cell conditions. Moreover, we specifically propose ways to integrate BCOs into retinal biosynthetic bacterial systems to improve the performance of retinal production.

## 1. Introduction

**Figure 1:**
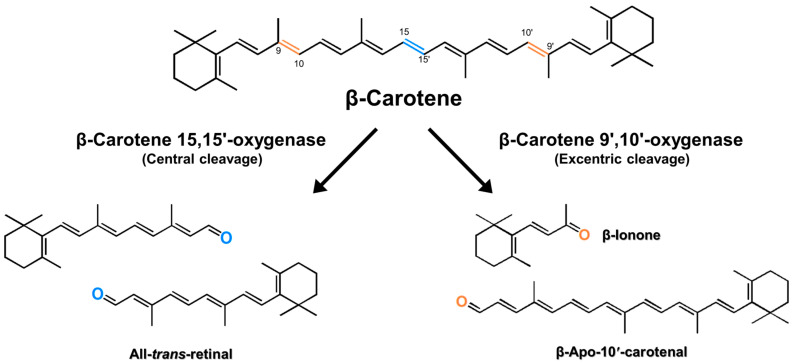
Enzymatic cleavage of β-carotene. Routes of formation of retinal (**left**) by β-carotene 15,15′-oxygenase (BCO1) and that of apo-carotenal and ionone (**right**) by β-carotene 9′,10′-oxygenase (BCO2).

Vitamin A, an essential nutrient for humans, is a fat-soluble natural precursor of signaling molecules and chromophores [1,2,3]. It belongs to the retinoid category, which is a class of chemical compounds that are either derivatives of vitamin A or are chemically related to it. Retinol, retinal, and retinoic acid are alcohol, aldehyde, and oxidized forms of vitamin A, respectively [4]. Vitamin A and its derivatives are used in pharmaceuticals, nutraceuticals, animal feed additives, functional foods, antioxidants, anti-aging, and anti-wrinkle cosmetic products [5,6,7]. Since humans cannot synthesize vitamin A, they obtain it only through diet [8]. Vitamin A can be synthesized from carotenoids, such as α-carotene, β-carotene, γ-carotene, β-cryptoxanthin, β-apo-carotenal, and β-apocarotenol, by carotenoid cleavage enzymes, including apo-carotenoid oxygenase (ACO), viviparous14 (VP14), neither inactivation nor after potential B (NinaB), and β-carotene oxygenase (BCO) [9,10]. Carotenoid cleavage enzymes constitute a family of enzymes that can metabolize carotenoids. They are classified into central cleavage enzymes and eccentric cleavage enzymes based on the position of asymmetric cleavage site. BCOs, which are known to play a key role in metabolizing carotenoids in vertebrates, are further classified into two types, namely β-carotene 15,15′-oxygenase (BCO1; EC 1.13.11.63) and β-carotene 9′,10′-oxygenases (BCO2; EC 1.14.99), based on the position of cleavage site on β-carotene (Figure 1). BCO1 is important as a retinoid producer, acting on the β-carotene 15,15′-double bond and producing two molecules of all-*trans*-retinal, while BCO2 acts on the β-carotene 9′,10′-double bond and produces ionone and apo-10′-carotenal (Figure 1), which can be further cleaved by BCO1 to produce other retinoids, such as retinaldehyde, retinol, and retinoic acid [8,11,12,13].

Retinoids, including retinal, retinol, and retinoic acid, are produced via chemical synthesis or enzymatic conversion. Chemical synthesis-based commercial production of retinal is performed by acidification/hydrolysis of retinal isomeric mixtures to produce retinol [4,14,15]. However, the chemical process has some disadvantages, such as involving multiple purification steps, by-product formation, and chemical waste formation [4]. On the other hand, the enzymatic conversion of β-carotene to retinal, using BCO, has some advantages, such as being an eco-friendly process and having reduced by-product generation due to the substrate-specificity of the enzyme. BCO is expressed in several tissues in humans, and its high enzymatic activity has been quantified in homogenates of liver and intestine [16,17,18,19,20]. In addition, BCOs from various organisms, such as animals, plants, and bacteria, have been reported as potential retinal producers (Table 1).

In this review, we summarize the biochemical properties and substrate specificities of BCOs and discuss the enzymatic bioconversion of β-carotene to retinal using these enzymes. Further, we propose several ways of efficient retinal production by bioengineering of BCOs and by optimizing the metabolic pathway responsible for retinal synthesis.

## 2. Biochemical Properties of BCO1 and BCO2

The characteristics of BCO1 and BCO2 from various organisms are summarized in Table 1. BCO1 derived from different sources (hereafter, BCO1s) are mainly found in animals, such as mammals [8,20,26,27,28,29,30,33,34,35], chicken [22,23,24,25], *Caenorhabditis elegans* [21,44], and uncultured marine bacterium [32]. BCO2 derived from different sources (hereafter, BCO2s) have been reported in a wide range of organisms, including animals [11,15,38,39,40], *Saccharomyces cerevisiae* [42], and plants such as apple [36], *Arabidopsis thaliana* [37], chrysanthemum [36], osmanthus [36], potato [41], rose [36], and saffron [43]. Most BCOs show optimal enzymatic activity at 28–40 °C and pH 7.4–8.0 (Table 1). However, human BCO1 shows optimal activity at a slightly acidic pH of 6.5 [27], and BCO2 from *S. cerevisiae* shows optimal activity at 45 °C [42].

The molecular weights of BCO1 subunits are approximately 60–65 kDa, and those of BCO2 subunits are approximately 50–64 kDa (Table 1). However, BCO1 derived from pig has a molecular weight of 156 kDa [34], and is larger in size than other BCOs. This result is not consistent with porcine genomes encoding BCO1 of about 65 kDa. On the contrary, the subunit of BCO1 derived from uncultured marine bacterium has the smallest molecular weight (38 kDa) among all BCOs, which is probably because BCO1 from uncultured marine bacterium is an enzyme belonging to bacterioopsin-related protein (Brp) or bacteriorhodopsin-related protein-like homolog protein (Blh), unlike other BCOs [32]. BCO1s show various associated forms, including monomers [23,27], dimers [21,32], and tetramers [24,30], whereas BCO2 from humans has been reported to exist only as a tetramer [15]. BCO1 from chicken exists as both a monomer and a tetramer, according to two different studies, even though it was expressed from the same gene. This might have been due to the synthesis of proteins by different host expression systems, such as baby hamster kidney (BHK) and *Escherichia coli* cells [23,24].

Metal ions play an important role in aiding the activities of several types of enzymes. Fe^2+^ has been suggested to affect the activity of BCO by binding to histidine residues at its active site [45,46]. Fe^2+^ has also been reported to have a strong impact on the activity of BCO1s and BCO2s. However, only BCO2 from *S. cerevisiae* showed an increase in activity with Ca^2+^ and a decrease in activity with Fe^2+^ [42]. Furthermore, this enzyme was not inhibited by EDTA treatment, suggesting that it is not a metalloprotein, unlike other BCOs.

## 3. Substrate Specificities of BCO1 and BCO62

BCOs exhibit various enzymatic properties with a large variation in activity depending on their origin, substrate, and the systems used for expression and purification. Therefore, based on quantitative comparison across BCOs, reported in different case studies, we have summarized the activities of BCO1s and BCO2s toward β-carotene and those with respect to various substrates, such as β-carotene, α-carotene, γ-carotene, lutein, β-cryptoxanthin, β-apo-4′-carotenal, β-apo-8′-carotenal, and β-apo-10′-carotenal, along with the relevant expression and purification methods (Table 2). Since the substrates of BCOs are generally very hydrophobic, their activity may depend on the substrate delivery methods such as detergent, solvent, and micellization. However, the difference in activity according to the substrate delivery method can be accurately compared under the same assay method. Therefore, in this review, only the values for activities are compared, except for a discussion of the substrate delivery methods.

The specific activity and catalytic efficiency (*k*_cat_/*K_m_*) of BCO1 derived from uncultured marine bacterium for β-carotene were the highest among all BCO1s [32] that were expressed in *E. coli*. BCO1 derived from chicken, and expressed in *E. coli*, showed a higher specific activity than that expressed in BHK cells, indicating that activity of BCO seems to be affected by enzyme expression conditions such as the expression vector and host cells. For example, under the pBAD system, BCO activity was approximately 20 times higher than under the pET system [24,25]. On the other hand, purified human-derived BCO1, expressed in SF9 cells showed a higher specific activity and catalytic efficiency than that expressed in *E. coli* cells [8,29]. BCO1s from chicken [24] and uncultured marine bacterium [32] had the highest *k*_cat_/*K_m_* values for β-carotene compared to the values for any other substrate, but BCO1 derived from human had the highest catalytic efficiency for lycopene [8]. BCO1 from uncultured marine bacterium had the highest *k*_cat_/*K_m_* value for β-carotene among all BCO1s [32]. Quantitative activities of purified BCO2s have only been reported in case of ferret [39], humans [15], and *S. cerevisiae* [42], which are fewer than reports on BCO1s. BCO2 from *S. cerevisiae* displayed the highest specific activity and *k*_cat_/*K_m_* for β-carotene than for other substrates [42].

Overall, BCOs derived from microorganisms, such as uncultured marine bacterium and *S. cerevisiae*, showed higher activity levels than those derived from animals, suggesting that they can be easily applied in biological engineering, with microbial cells as hosts, for the improvement of enzyme expression and performance in future.

## 4. Retinal Production by Biological Methods

Retinal is used by the food, cosmetics, and pharmaceutical industries. Its industrial production involves chemical synthesis via acid or base reduction, which has some disadvantages, such as use of expensive reagents, complex purification steps, generation of toxic chemical waste, and formation of undesired by-products [4]. Thus, efficient biological methods, using enzymes and microbial cells, for retinal production have been studied, considering that they are more cost-effective and environment-friendly than chemical methods [47,48,49,50].

Retinal production by enzymes or metabolically engineered cells is summarized in Table 3. BCO1 from various sources, such as mouse [51], human [52], chicken [24], fruit fly [53], uncultured marine bacterium [32], and pig [34], have been used for the enzymatic production of retinal. Among those, uncultured marine bacterium-derived BCO1 showed the highest retinal production through optimization of reaction conditions such as type and concentration of solvent and detergent for the formation of micelles, and concentrations of enzyme and β-carotene. This enzyme, which produced 181 mg/L retinal, was produced from 350 mg/L β-carotene at pH 8.0, 40 °C, 2.4% Tween 20 (*w*/*v*), and 15 U/mL enzyme with a conversion yield of 52% and a productivity of 9.1 mg/L/h [14]. On the other hand, chicken-derived BCO had the highest conversion yield (60%) at pH 8.0 and 37 °C in the presence of 0.64 mg/mL Tween80 and 0.2 U/mL enzyme, which produced 3.2 mg/L retinal production from 5.37 mg/L β-carotene with a productivity of 1.06 mg/L/h [24].

Retinal has also been produced by metabolically engineered cells such as *E. coli* and *S. cerevisiae*. To produce retinal using metabolically engineered *E. coli*, the methylerythritol 4-phosphate (MEP) pathway and mevalonate (MVA) pathway can be adopted with BCOs. Lee et al. produced up to 600 mg/L retinal using metabolically engineered *E. coli* [54], which is the highest retinal production among the engineered *E. coli* cells. Additionally, Zhang et al. developed a “plug-n-play” platform, which can simultaneously produce retinol, retinal, and various apocarotenoids by modifying apocarotenoid genetic module [58]. However, *E. coli* might not be the most desirable host cell for industrial production of retinal, owing to possible endotoxin contamination of the final product. The other candidate host, *S. cerevisiae*, is relatively superior for the industrial production of retinal due to easy genetic manipulation, robust compatibility for large-scale fermentation, and expression of the MVA pathway, which has a relatively high flux towards isoprenoids. Recently, Sun et al. developed an engineered *S. cerevisiae* with BCO1 to produce vitamin A, which consisted of retinal and retinol, via a xylose fed-batch fermentation [60]. The engineered *S. cerevisiae* produced 2094 mg/L retinal from xylose via β-carotene biosynthetic pathway with a productivity of 13.1 mg/L/h, which is the highest production of retinal in vitamin A synthesis [60], indicating that biological engineering systems can be used for sustainable bioprocesses for the industrial production of retinal using BCOs.

## 5. Proposed Roles of Amino Acids in BCOs

**Figure 2:**
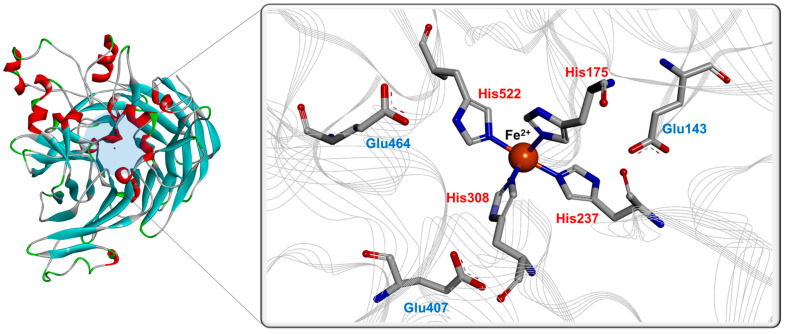
Active site in molecular model of BCO1. The three-dimensional structure was adopted from *C. elegans* BCO1 (PDB no. 7WH0).

To identify the critical amino acid residues involved in BCO activity, structural analyses using homology modeling and site-directed mutagenesis have been performed. Recently, crystal structures of enzymes annotated as putative BCOs from *C. elegans* were reported; however, the enzymes did not exhibit any activity [21]. Moreover, a new crystal structure of the carotenoid cleavage enzyme from *Candidatus Nitrosotalea devanaterra* was determined recently [61]. However, this enzyme showed no activity toward bicyclic carotenoids such as β-carotene. Since the crystal structure of the active BCO enzyme has not been determined yet, a homology model of BCO1 was built based on the ACO crystal structure [45,62]. BCOs are metal-dependent enzymes, and most of them prefer Fe^2+^ for their enzymatic activity (except BCO1 from *S. cerevisiae* that prefers Ca^2+^). Fe^2+^ is coordinated by four strictly conserved His residues with three Glu residues, which involve the formation of second coordination sphere via hydrogen bonds (Figure 2, show the three-dimensional structure of BCO1 from *C. elegans*).

**Figure 3:**
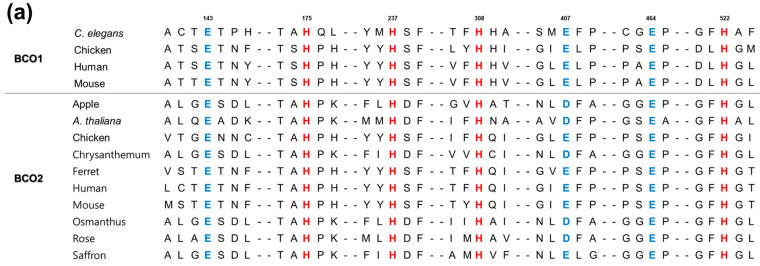

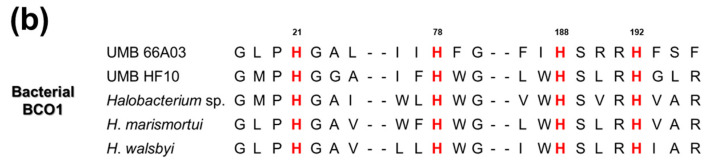
Sequence alignments of BCOs. (**a**) Alignment of BCOs from mammals, chicken, *C. elegans*, and plants. The numbers for amino acids are represented based on BCO1 from *C. elegans*. (**b**) Alignment of bacterial BCOs, adapted from previous report [32]. The numbers for amino acids are represented based on BCO1 from uncultured marine bacterium 66A03. Abbreviation: UMB, uncultured marine bacterium. The UniProt numbers for BCOs are as follows: *C. elegans*, Q9U2E4; chicken, Q9I993; human, Q9HAY6; mouse, Q9JJS6; apple, A9Z0V8; *A. thaliana*, O65572; chicken, E1C8E0; chrysanthemum, B0FLM7; ferret, Q6QT07; human, Q9BYV7; mouse, Q99NF1; osmanthus, B0FLM9; rose, B0FLM8; saffron, A0SE36; UMB 66A03, Q4PNI0; UMB HF10, A4GIC0; *Halobacterium* sp., Q9HNE6; *H. marismortui*, Q5V0N2; and *H. walsbyi*, Q18DH3. Residues involved in the metal coordination and the formation of second coordination sphere via hydrogen bonds are represented by the red and blue colors, respectively.

The critical residues in carotenoid cleavage enzymes are His and Glu, which are conserved the most of carotenoid cleavage enzymes [61]. The His and Glu residues in the predicted active site of BCO were mutated, resulting in loss of enzyme activity [45]. The four His residues, as metal-binding residues, were all conserved in the reported BCOs (Figure 3a), and in BCO1 from uncultured marine bacterium they were absolutely conserved as those of putative bacterial BCOs from *Halobacterium* sp., *Halobacterium marismortui*, and *Halobacterium walsbyi* [32] (Figure 3b). Kim et al. conducted site-directed mutagenesis for the His into Ala residues of uncultured marine bacterium (specific strain No. 66A03) in order to identify the role of these critical residues. Replacements of His21, His78, His188, and His192 by Ala showed significantly decreased activities (to below 5%) compared to the activity of wild-type enzyme [32]. On the other hand, one of the three Glu residues is replaced by Asp in some BCO2s from plants, such as apple, *A*. *thaliana*, chrysanthemum, osmanthus, and rose (Figure 3a), indicating that negatively charged amino acids may be involved in BCO activity.

The roles of other amino acid residues in BCO1 were suggested by Poliakov et al. They conducted mutagenesis to determine the role of aromatic residues (especially Tyr), which are the nearby acidic residues such as Glu, using the homology model of BCO1 from mouse that was built based on ACO structure [62]. Replacements of Tyr235 by Leu and Tyr326 by Glu showed decreased enzyme activity toward β-carotene, and the two Tyr residues were proposed to possibly be involved in enzyme stabilization via cation-π interactions between them, with the β-carotene cationic intermediate playing an important role in the reaction mechanism [62].

Despite the efforts to understand the roles of amino acid residues of BCO, only those involved in metal coordination and BCO stabilization have been reported so far. For industrial applications of retinal biosynthesis, BCOs should be further engineered, through the determination of new crystal structures and identification of critical residues for increased activity.

## 6. Future Perspectives of BCO in Synthetic Bacteria

**Figure 4:**
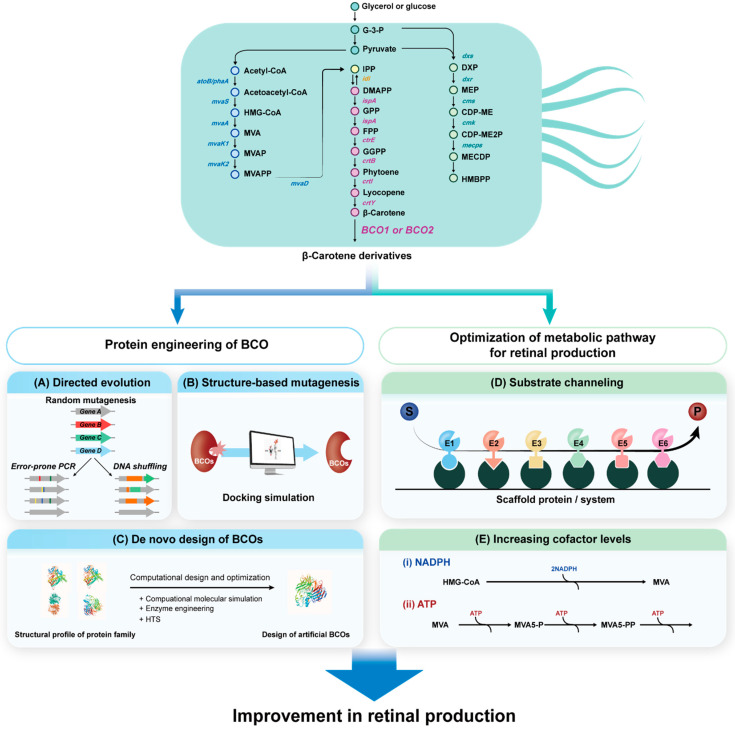
Strategies for the improvement of retinal production via protein engineering of BCO by directed evolution (**A**), structure-based mutagenesis (**B**), d novo design of BCOs (**C**), optimization of metabolic pathway (MVA pathway, left; MEP pathway, right) for retinal production (top) through substrate channeling system (**D**), and co-factor regeneration system to increase the co-factor levels (**E**). Abbreviation for the compounds: G-3-P, glucose-3-phosphate; HMG-CoA, 3-hydroxy-3-methylglutaryl CoA; MVA, mevalonic acid; MVAP, phosphomevalonate; MVAPP, diphosphomevalonate; IPP, isopentenyl pyrophosphate; DMAPP, dimethylallyl pyrophosphate; GPP, geranyl pyrophosphate; FPP, farnesyl pyrophosphate; GGPP, geranylgeranyl pyrophosphate; DXP, 1-deoxy-d-xylulose-5-phosphate; MEP, methylerythritol phosphate; CDP-ME, 4-diphosphocytidyl-2-*C*-methyl-d-erythritol; CDP-ME2P, 4-diphosphocytidyl-2-*C*-methyl-d-erythritol 2-phosphate; MECDP, 2*C*-methyl-d-erythritol 2,4-cyclodiphosphate; HMBPP, 1-hydroxy-2-methyl-2-butenyl 4-diphosphate. The genes encode the following enzymes: *atoB/phaA*, acetoacetyl-CoA synthase; *mvaS*, HMG-CoA synthase; *mvaA*, HMG-CoA reductase; *mvaK1*, mevalonate kinase; *mvaK2*, phosphomevalonate kinase; *mavD*, mevalonate-5-diphosphate decarboxylase; *idi*, IPP isomerase; *ispA*, FPP synthase; *crtE*, GGPP synthase, *crtB*, phytoene synthase; *crtI*, phytoene desaturase; *crtY*, lycopene cyclase; *BCO1*, β-carotene 15,15′-oxygenase; *BCO2*, β-carotene 9,10′-oxygenase; *dxs*, 1-deoxy-d-xylulose-5-phosphate synthase; *dxs*, 1-deoxy-d-xylulose-5-phosphate reductoisomerase; *cms*, 4-diphosphocytidyl-2C-methyl-d-erythritol synthase, *mecps*, 2C-methyl-d-erythriol-2,4-cyclodiphosphate synthase. Dashed arrow indicates multi-enzymatic steps.

Many challenges in retinal production have been addressed using metabolically engineered *E. coli* cells. Several aspects have been considered for the efficient production of carotenoid, which can be used as a substrate for BCOs, from glucose or glycerol via the endogenous MEP pathway and exogenous MVA pathway in metabolically engineered cells [63,64,65,66,67,68]. However, studies on the engineering of BCOs, which are crucial enzymes for catalyzing the last step of the retinal biosynthetic pathway, are still lacking. As mentioned above, BCOs have been successfully derived from animals, plants, and a few bacteria (Table 1), and need to be engineered to maximize the conversion of β-carotene to retinal. The catalytic properties of native BCOs can be improved via directed evolution, structure-based mutagenesis, and de-novo designing of the protein structure [69] (Figure 4). Directed evolution techniques require large libraries and efficient screening to select BCOs with the desired activity (Figure 4A). The DNA shuffling method can be used for genetic engineering of BCOs from different organisms to alter the conformation or orientation of the entire enzyme, including that of the active site, and obtain BCOs capable of efficient production of retinal. The library for DNA shuffling can be easily expanded by BCO genes from various organisms, which can efficiently obtain engineered BCOs with the desired activity [70] (Figure 4A). Structure-based mutagenesis, based on molecular modelling and rational considerations with respect to known structures of BCOs, can also be used to change the amino acid residues of the catalytic site (Figure 4B). Recently, advances in synthetic biology have enabled de-novo design and synthesis of proteins that have new functions and activities. Since the known BCO, expressed by microbial cells, lacks efficient and high-turnover retinal production, de-novo design will be a novel alternative to enhance the industrial production of retinal (Figure 4C). However, the designed components should be biocompatible with natural biochemical processes and pathways, and not interfere with cellular function [71].

Another engineering strategy for improving retinal production is the metabolon system, which can be considered for the optimization of upstream metabolic pathway for carotenoid bioconversion. Metabolons, which are complexes that use scaffold proteins to form multi-enzyme catalysts, have recently attracted much attention due to their ability for synergistic and simultaneous action of different types of enzymes in metabolic pathways (Figure 4D). They enhance metabolic flux using pushing and pulling mechanisms, which can also be applied to carotenoid production. Moreover, metabolons reduce metabolic burden by increasing the efficiency of intermediate transfer, in turn reducing intermediate toxicity and increasing product yield. Recently, affibody scaffolds formed via noncovalent interactions have been applied to improve sesquiterpene production in *S. cerevisiae* [72], and zinc-finger motifs on DNA scaffold systems have been used to improve the metabolic efficiency of the lycopene biosynthesis pathway [73]. The SpyTag/SpyCatcher system and its derivatives, which are covalently conjugated systems, have become the most versatile and widely used enzyme-assembly systems for diverse applications [74,75]. The design of cascade reactions using scaffolds can be applied to the MEP pathway for the efficient production of retinal. Furthermore, increasing the level of co-factors such as ATP and NADPH, which play important roles as donors of phosphate and electrons, respectively, are crucial for metabolic pathways, so that they can be used to increase the efficiency of enzymes involved in the MVA pathway for carotenoid production [76]. Levels of NADPH can be increased by enhancing metabolic flux through the pentose phosphate pathway via knock out of genes encoding glucose 6-phosphate isomerase and PEP carboxylase [77]. Increasing and replenishing the co-factor pools (NADPH, ATP, etc.) can be an effective strategy to enhance the rate of retinal biosynthesis, since they form the major intracellular energy source for many biological reactions (Figure 4E).

Finally, the construction and culture of BCO-driven retinal-producing strains are still in their early stages. Thus, further optimization of fermentation operations (such as fed-batch cultivation) and media, and improvement of strains, may increase the current titer of retinal produced by *E. coli*. Advances in enzymatic engineering and metabolically engineered cells have already provided many advantages in industrial processes, but there are still few attempts to utilize BCOs for the retinal production. We expect future studies in the design and engineering of BCOs, and the associated pathways will accelerate improvements in retinal production on an industrial scale process.

## 7. Conclusions

BCOs are important biocatalysts in the production of retinal. The current review summarizes the biochemical properties and substrate specificities of BCOs and the production of retinal using these enzymes. Retinal can be produced from metabolically engineered cells via enhancing the MVA or MEP pathway. BCOs are the final enzymes in retinal production. Thus, they should be engineered to improve their enzymatic performance. In this review, we propose several ways of enzyme engineering of BCOs and optimization of the metabolic pathway responsible for retinal synthesis. Bioengineering of BCOs can be the key to high-level retinal production. Furthermore, engineered cells, and an improved via synthetic biological approach, should be developed for the industrial production of retinal.

## Figures and Tables

**Table 1:** Biochemical properties of BCO1 and BCO2.

Type /Organism	Accession No.	Optimum Temperature (°C)	Optimum pH	Molecular Weight (kDa)		Associated Form	Metal Ion	References
				Subunit	Native			
BCO1								
*Caenorhabditis elegans^R^*	Q9U2E4	37	7.5	–	–	dimer	–	[21]
Chicken*^R^*	Q9I993	–	–	60	–	–	–	[22]
Chicken*^R^*		37	8.0	60	≈50	monomer	–	[23]
Chicken*^R^*		37	8.0	60	240	tetramer	Fe^2+^	[24]
Chicken*^R^*		37	–	64	–	–	–	[25]
Guinea pig*^N^*	–	37	8.5	–	–	–	Fe^2+^	[26]
Human*^R^*	Q9HAY6	37	6.5	65	–	monomer	Fe^2+^	[27]
Human*^R^*		37	8.0	–	–	–	–	[28]
Human*^R^*		37	8.0	–	–	–	–	[8]
Human*^R^*		28	7.6	–	–	–	–	[29]
Human*^R^*		37	7.5–8.0	≈64	≈230	tetramer	Fe^2+^	[30]
Human*^R^*		30	7.0	–	–	dimer	Fe^2+^	[31]
Uncultured marine bacterium*^R^*	Q4PNI0	40	8.0	32	64	dimer	Fe^2+^	[32]
Mouse*^R^*	Q9JJS6	37	8.0	65	–	–	Fe^2+^	[33]
Pig*^N^*	–	37	8.0	156	–	–	–	[34]
Rat*^N^*	–	–	7.5–8.0	62	62	monomer	–	[35]
Rat*^N^*	–	37	8.0	–	–	–	–	[20]
BCO2								
Apple*^R^*	A9Z0V8	30	≈7.4	–	–	–	–	[36]
*Arabidopsis thaliana^R^*	O65572	30	≈7.4	–	–	–	–	[37]
Chicken*^R^*	E1C8E0	37	8.0	64	–	–	Fe^2+^	[38]
Chrysanthemum*^R^*	B0FLM7	30	≈7.4	–	–	–	–	[36]
Ferret*^R^*	Q6QT07	37	8.5	≈64	–	–	Fe^2+^	[39]
Human*^R^*	Q9BYV7	–	–	64	–	–	–	[40]
Human*^R^*		37	8.0	58	240	tetramer	Fe^2+^	[15]
Mouse*^R^*	Q99NF1	37	8.0	–	–	–	–	[11]
Mouse*^R^*		30	7.0	–	–	dimer	Fe^2+^	[31]
Osmanthus*^R^*	B0FLM9	30	≈7.4	–	–	–	–	[36]
Potato*^N^*	–	28	7.8	–	–	–	Fe^2+^	[41]
Rose*^R^*	B0FLM8	30	≈7.4	–	–	–	–	[36]
*Saccharomyces cerevisiae^N^*	–	45	8.0	50	–	–	Ca^2+^	[42]
Saffron*^R^*	A0SE36	30	–	–	–	–	–	[43]

*^N^* native enzyme; *^R^* recombinant enzyme.

**Table 2:** Summary of substrate specificities of BCOs.

Type /Organism	Expression	Substrate	Specific Activity (nmol/min/mg)	*k*_cat_/*K_m_* (min^−1^ mM^−1^)	References
BCO1					
Chicken	pBAD system in *E. coli* cells*^P^*	β-carotene	6.8		[25]
Chicken	SFV system in BHK cells*^H^*	β-carotene	0.04		[22]
Chicken	SFV system in BHK cells	β-carotene	0.04		[23]
Chicken	pET system in *E. coli* cells*^H^*	β-carotene	0.32	64.0	[24]
		α-carotene		1.3	
		γ-carotene		0.7	
		β-cryptoxanthin		7.1	
		β-apo-4′-carotenal		4.9	
		β-apo-8′-carotenal		6.0	
Guinea pig	Extraction from intestine*^P^*	β-carotene	0.03		[26]
		lutein	0.02		
		β-apo-10′-carotenal	0.02		
Human	expression in SF9 cells	β-carotene	18.5		[29]
Human	expression in SF9 cells	β-carotene		93.0	[30]
		β-cryptoxanthin		19.0	
Human	pET system in *E. coli* cells*^H^*	β-carotene	2.20	6.10	[8]
		α-carotene		1.81	
		β-cryptoxanthin		1.43	
		lycopene		8.85	
		β-apo-8′-carotenal		2.72	
Uncultured marine bacterium	pET system in *E. coli* cells*^H^*	β-carotene	45.0	97.0	[32]
		α-carotene		3.60	
		γ-carotene		0.55	
		β-cryptoxanthin		28.0	
		β-apo-4′-carotenal		4.20	
Mouse	pBAD system in *E. coli* cells*^H^*	β-carotene		0.39	[33]
Rat	extraction from intestine	β-carotene	0.012		[20]
BCO2					
Chicken	pET system in *E. coli* cells*^H^*	β-carotene		0.49	[38]
Human	pET system in *E. coli* cells*^H^*	β-carotene	80.0		[15]
*S. cerevisiae*	extraction from microbial cells	β-carotene	380.0	77.0	[42]
		α-carotene		49.0	
		lutein		10.0	

*^P^* partially purified enzyme; *^H^* purification of his-tagged protein.

**Table 3:** Production of retinal using BCO1 and metabolically engineered cells.

Biocatalyst	Source/Host	Substrate (mg/L)	Product (mg/L)	Reaction Time (h)	Conversion Yield (%)	Productivity (mg/L/h)	References
BCO1	Mouse	200	72	2	36	4.8	[51]
	Human	200	98	16	49	6.13	[52]
	Chicken	5.37	3.2	16	60	1.06	[24]
	Fruit fly	0.17	0.13	2	18	0.065	[53]
	Uncultured Marine bacterium	350	181	20	52	9.1	[14]
	Pig	44	14.65	20	33.3	0.73	[34]
Metabolically engineered cells	*E. coli*	–	600	33	–	18	[54]
	*E. coli*	–	67	72	–	0.93	[55]
	*E. coli*	–	7	72	–	0.1	[56]
	*E. coli*	–	2.26	36	–	0.062	[57]
	*E. coli*	–	5.1	50	–	0.102	[58]
	*S. cerevisiae*	–	221.37	20	–	11.065	[59]
	*S. cerevisiae*	–	2094	160	–	13.1	[60]
